# Inhibitory Effects of Gyeji-Tang on MMP-9 Activity and the Expression of Adhesion Molecules in IL-4- and TNF-α-Stimulated BEAS-2B Cells

**DOI:** 10.3390/plants10050951

**Published:** 2021-05-10

**Authors:** Yu Jin Kim, Woo-Young Jeon, Youn-Hwan Hwang, Mee-Young Lee

**Affiliations:** Herbal Medicine Research Division, Korea Institute of Oriental Medicine, 1672 Yuseong-daero, Yuseong-gu, Daejeon 34054, Korea; jinjin0228@kiom.re.kr (Y.J.K.); ssamggun85@kiom.re.kr (W.-Y.J.)

**Keywords:** Gyeji-tang, airway inflammation, BEAS-2B cells, eotaxins, RANTES, matrix metalloproteinase-9, UPLC-DAD-MS/MS

## Abstract

Gyeji-tang (GJT), a traditional herbal formula composed of five herbal medicines, is commonly used to treat the common cold, exogenous febrile disease, fever and headaches in Korea, China and Japan. Although various pharmacological activities of GJT have been reported in several studies, the effect of GJT water extract (GJTWE) on airway inflammation has not yet been investigated. This study aimed to evaluate the effects of GJTWE on airway inflammation-related factors using human bronchial epithelial BEAS-2B cells, and to identify the phytochemicals in GJTWE by ultra-performance liquid chromatography-diode array detector-tandem mass spectrometry (UPLC-DAD-MS/MS) analysis. GJTWE significantly decreased the production of chemokines, including eotaxin-3, eotaxin-1, regulated on activation normal T-cell expressed and secreted (RANTES), and matrix metalloproteinase-9, and the expression of the adhesion molecules, intercellular adhesion molecule-1 and vascular cell adhesion molecule-1, in interleukin-4 + tumor necrosis factor-α (IT)-stimulated BEAS-2B cells. In the UPLC-DAD-MS/MS analysis, 21 phytochemicals, including six flavonoids, two chalcones, five terpenoids, six phenolics, one phenylpropanoid and one coumarin, were identified in GJTWE. The findings suggested that GJTWE might exhibit anti-inflammatory effects on airway inflammation by regulating the expression of inflammatory response-related factors in IT-stimulated BEAS-2B cells; further studies are required to determine the bioactive compounds involved in the inhibition of airway inflammation.

## 1. Introduction

Airway inflammation is an important factor in the pathogenesis of obstructive airway diseases, such as asthma and chronic obstructive pulmonary disease [1]. The inflammatory response of the airway epithelium, mediated by the increased expression of chemokines, cytokines, inflammatory enzymes, and adhesion molecules, involves the recruitment, activation, and infiltration of inflammatory cells, along with the tissue remodeling of airways [1,2]. The bronchial epithelium plays a central role in regulating the airway inflammatory response [3]. The human bronchial epithelial BEAS-2B cells stimulated by interleukin (IL)-4, tumor necrosis factor (TNF)-α, or lipopolysaccharide have been reported to secrete chemokines and cytokines such as eotaxin-1, eotaxin-3, regulated on activation normal T-cells expressed and secreted (RANTES), and IL-8, which contribute to airway inflammation [4,5,6]. In addition, BEAS-2B cells treated with IL-4 and TNF-α increase the expression of matrix metalloproteinase (MMP)-9, an important proteolytic enzyme that induces bronchial remodeling in asthma [4]. Tissue remodeling in the airways may cause airway hyperresponsiveness, and may reduce the reversibility of the airflow obstruction in asthma [5]. The accumulation of leukocytes in the inflamed sites of the airways is a hallmark of asthma, and the infiltration of inflammatory cells occurs through the expression of adhesive molecules that regulate the adhesion of leukocytes and epithelial cells [7]. The expression of adhesion molecules, intercellular adhesion molecule-1 (ICAM-1) and vascular cell adhesion molecule-1 (VCAM-1) has been reported to be induced by cytokines in human bronchial epithelial cells [8]. Therefore, suppressing the production, expression, and activation of cytokines, chemokines, inflammatory enzymes, and adhesion molecules in bronchial epithelial cells may help relieve airway inflammation.

Gyeji-tang (GJT), alternatively called Gui-Zhi-Tang in China and Keishi-to in Japan, is one of the traditional herbal formulae in Shang-Han-Lun, and has been widely used to treat the common cold, exogenous febrile disease, fever, headache, and inflammation [9,10,11]. GJT is comprised of five herbs: *Cinnamomum cassia* Blume (twig), *Paeonia lactiflora* Pall. (root), *Glycyrrhiza uralensis* Fisch. (root and rhizome), *Zingiber officinale* Rosc. (rhizome), and *Ziziphus jujuba* Mill. (fruit). The pharmacological effects of GJT had been reported previously. It exhibits antipyretic effects by attenuating bradykinin-induced prostaglandin E_2_ (PGE_2_) release from rabbit astrocytes [11], anti-inflammatory effects by blocking extracellular signal-regulated kinase (ERK) and nuclear factor kappa-B (NF-κB) signaling pathways in lipopolysaccharide-stimulated RAW 264.7 cells [12], and immunosuppressive effects by inhibiting IL-2 production in murine spleen cells [13]. In addition, animal studies have demonstrated the administration of GJT to ameliorate impairments in sociability, spatial attention, and fear memory deficits by restoring neuronal functions [14], and that it is effective in chronic pancreatitis caused by pancreatic ischemia [15]. Despite the various reported pharmacological activities of GJT, their effects on airway inflammation have not yet been investigated. The present study aimed to explore the effects of GJT water extract (GJTWE) on airway inflammation-related factors, such as eotaxin-3, eotaxin-1, RANTES, MMP-9, ICAM-1, and VCAM-1, in the human bronchial epithelial BEAS-2B cell line. In addition, the phytochemicals of GJTWE were identified using ultra-performance liquid chromatography-diode array detector-tandem mass spectrometry (UPLC-DAD-MS/MS) analysis.

## 2. Results

### 2.1. UPLC-DAD-MS/MS Analysis of GJTWE

**Figure 1:**
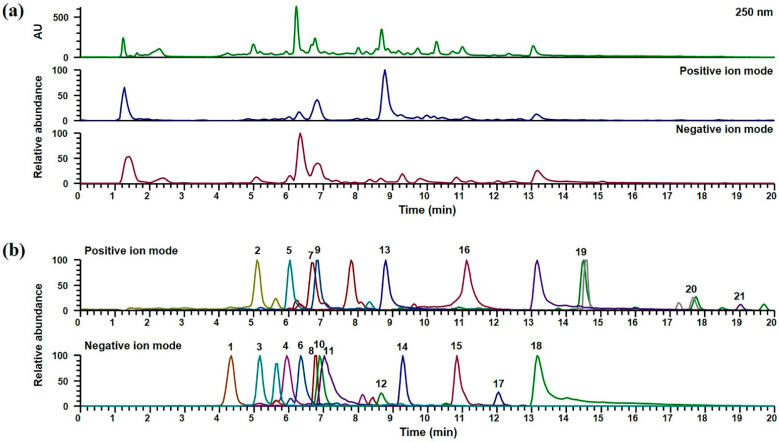
UPLC-DAD-MS/MS chromatograms of GJTWE. UV and base peak chromatograms (**a**) and extracted ion chromatograms in the positive and negative ion modes (**b**) of the identified phytochemicals. 1: protocatechuic acid; 2: protocatechualdehyde; 3: catechin; 4: schaftoside; 5: albiflorin; 6: paeoniflorin; 7: isovitexin; 8: liquiritin apioside; 9: syringaldehyde; 10: liquiritin; 11: 1;2;3;4;6-*O*-pentagalloylglucose; 12: isoliquiritin; 13: coumarin; 14: liquiritigenin; 15: benzoylpaeoniflorin; 16: cinnamaldehyde; 17: isoliquiritigenin; 18: glycyrrhizin; 19: 6-gingerol; 20: 6-shogaol; 21: glycyrrhetinic acid.

In order to identify the phytochemicals in GJTWE, UPLC-DAD-MS/MS analysis was performed. The chromatographic separation of the compounds in GJTWE was achieved on an Acquity BEH C_18_ column (100 × 2.1 mm, 1.7 µm, Waters) at 40 °C for 20 min, with mobile phases consisting of 0.1% (*v*/*v*) formic acid in water and acetonitrile. Both the positive and negative ion modes were used to acquire the MS spectra of each compound. In total, 21 compounds, namely protocatechuic acid, protocatechualdehyde, syringaldehyde, cinnamaldehyde, and coumarin from C. cassia [16]; albiflorin, paeoniflorin, benzoylpaeoniflorin, 1,2,3,4,6-O-pentagalloylglucose, and catechin from P. lactiflora [17]; schaftoside, liquiritin apioside, liquiritin, liquiritigenin, isoliquiritin, isoliquiritigenin, glycyrrhizin, and glycyrrhetinic acid from G. uralensis [17]; 6-gingerol and 6-shogaol from Z. officinale [18]; and isovitexin from Z. jujuba [19], were identified. The detailed MS data are listed in Table 1. The retention times, precursor ions, and MS/MS fragments of each compound were compared to those of the reference standards. Flavonoids and chalcones were more suitably ionized in the negative ion mode, while most terpenoids, coumarins, and other phenolic compounds were detected in the positive ion mode. The UV pattern at 250 nm and the base peak chromatograms in the positive and negative ion modes of GJTWE are shown in Figure 1a, and the extracted ion chromatograms for each compound are presented in Figure 1b.

### 2.2. Cytotoxicity of GJTWE in BEAS-2B Cells

**Figure 2:**
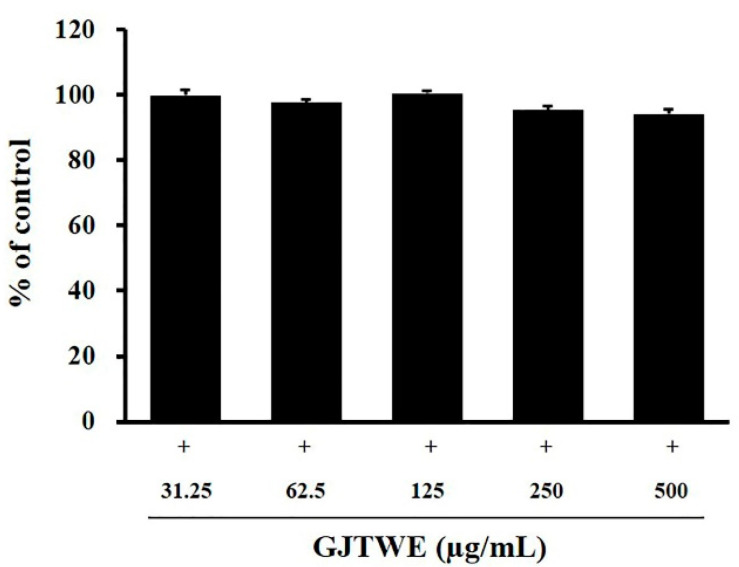
Cytotoxic effects of GJTWE in BEAS-2B cells. BEAS-2B cells were seeded into 96-well plates and treated with various concentrations (31.25, 62.5, 125, 250, and 500 μg/mL) of GJTWE for 24 h. The cell viability was assessed using a CCK-8 kit. The values are expressed as the mean ± SEM.

In order to determine the cytotoxicity of the test materials in BEAS-2B cells, the latter were exposed to various concentrations of GJTWE for 24 h. The cell viability was measured subsequently using a cell counting kit (CCK)-8 assay. GJTWE did not produce any significant cytotoxic effect at any concentration. Non-toxic concentrations (≤500 μg/mL) of the test materials were used for the subsequent experiments (Figure 2).

### 2.3. Effect of GJTWE on Chemokine Oroduction

**Figure 3:**
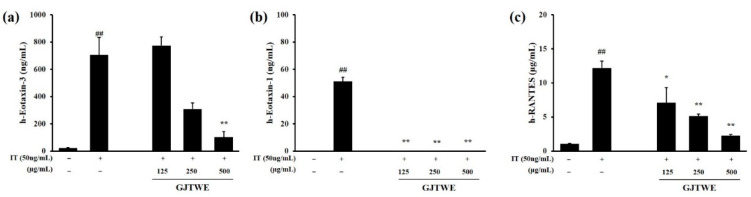
Effects of GJTWE on the production of chemokines in BEAS-2B cells. The cells were pretreated with GJTWE (125, 250, and 500 μg/mL) and then co-stimulated with IL-4 + TNF-α (IT, 50 ng/mL) for 48 h. The levels of h-eotaxin-3 (**a**), h-eotaxin-1 (**b**), and h-RANTES (**c**) released into the culture medium were assessed using commercially available ELISA kits. The values are expressed as the mean ± SEM. ^##^ *p* < 0.01 versus vehicle-treated cells and ^*^ *p* < 0.05 or ^**^ *p* < 0.01 versus IT-treated cells.

The effects of GJTWE on eotaxin-3, eotaxin-1, and RANTES production were assessed in IT-stimulated BEAS-2B cells. The production of eotaxin-3, eotaxin-1, and RANTES was significantly increased upon IL-4 + TNF-α (IT) stimulation compared to that in the vehicle-treated cells (*p* < 0.01). However, GJTWE significantly decreased the production of chemokines, such as eotaxin-3, eotaxin-1, and RANTES, in a dose-dependent manner (*p* < 0.05, *p* < 0.01, Figure 3a–c).

### 2.4. Effect of GJTWE on MMP-9 Activity

**Figure 4:**
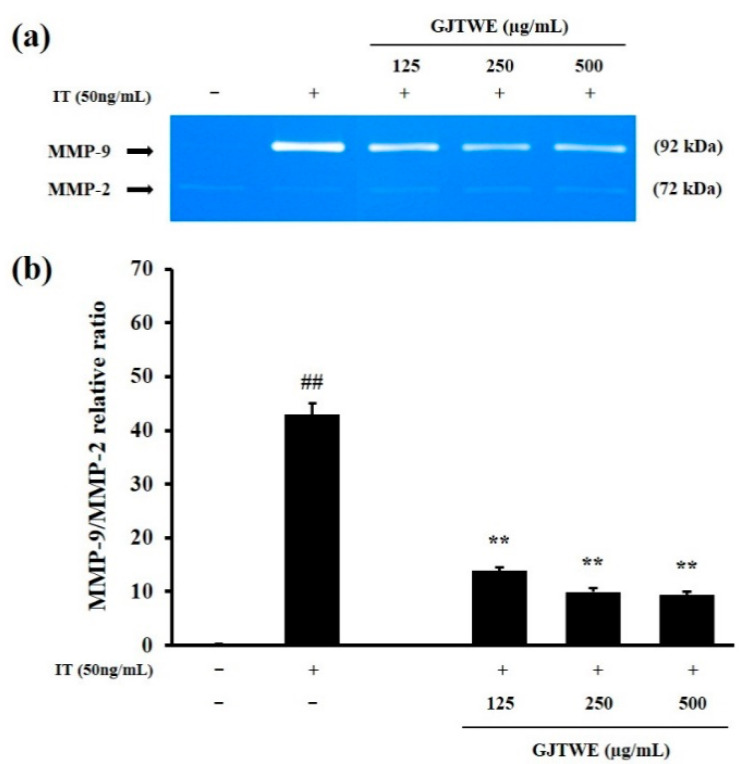
Effects of GJTWE on the activity of MMP-9 in BEAS-2B cells. The cells were pretreated with GJTWE (125, 250, and 500 μg/mL) and then co-stimulated with IL-4 + TNF-α (IT, 50 ng/mL) for 48 h. The cell supernatants were loaded for gelatin zymography. Representative photographs of the MMP-9 activity (**a**) and MMP-9/MMP-2 band intensities (**b**) are shown. The values are expressed as the mean ± SEM. ^##^ *p* < 0.01 versus vehicle-treated cells and ** *p* < 0.01 versus IT-treated cells.

In order to identify the regulator of inflammatory processes, we determined the activities of MMP-9 and MMP-2 in IT-stimulated BEAS-2B cells. As shown in Figure 4a, MMP-9 activity was remarkably increased by the IT treatment. However, GJTWE greatly reduced the MMP-9 activity in a dose-dependent manner. The relative ratio of MMP-9/MMP-2 was significantly increased in the IT-treated cells compared to that in the vehicle-treated cells (*p* < 0.01). In contrast, GJTWE significantly decreased the relative ratio of MMP-9/MMP-2 compared to that in the IT-treated cells (*p* < 0.01, Figure 4b).

### 2.5. Effect of GJTWE on the Expression of Adhesion Molecules

**Figure 5:**
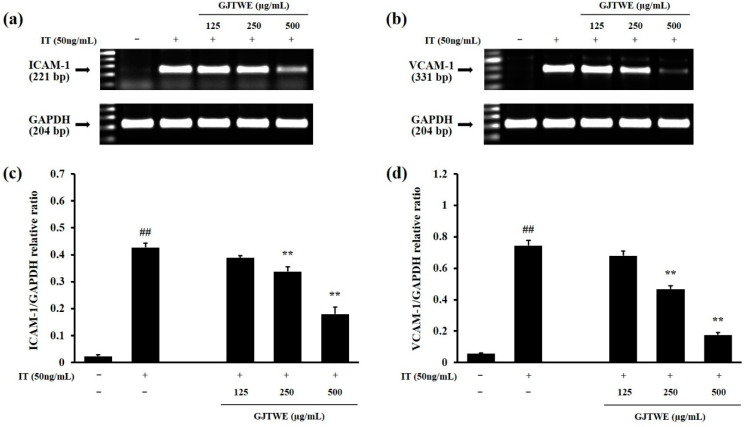
Effects of GJTWE on the expression of adhesion molecules in BEAS-2B cells. The cells were pretreated with GJTWE (125, 250, and 500 μg/mL) and then co-stimulated with IL-4 + TNF-α (IT, 50 ng/mL) for 48 h. The total RNA was isolated, and RT-PCR was performed in order to analyze the expression of ICAM-1 and VCAM-1 at the mRNA level. Representative photographs of the ICAM-1 (**a**) or VCAM-1 (**b**) expression, and the band intensities of ICAM-1/GAPDH (**c**) or VCAM-1/GAPDH (**d**) are shown. The values are expressed as the mean ± SEM. ^##^ *p* < 0.01 versus vehicle-treated cells and ** *p* < 0.01 versus IT-treated cells.

In order to identify the indicators of inflammatory responses, we determined the ICAM-1 and VCAM-1 expression in the IT-stimulated BEAS-2B cells. As shown in Figure 5a,b, the ICAM-1 and VCAM-1 expression was remarkably increased by the IT treatment. However, GJTWE dramatically decreased the ICAM-1 and VCAM-1 expression in a dose-dependent manner. The relative ratios of ICAM-1/glyceraldehyde-3-phosphate dehydrogenase (GAPDH) and VCAM-1/GAPDH were significantly increased in the IT-treated cells compared to the vehicle-treated cells (*p* < 0.01). In contrast, GJTWE significantly reduced the relative ratios of ICAM-1/GAPDH and VCAM-1/GAPDH compared to that in the IT-treated cells (*p* < 0.01, Figure 5c,d).

## 3. Discussion

The human bronchial epithelial cell line, BEAS-2B, has been previously used to examine cytokine- or endotoxin-associated airway inflammation, which may induce allergic asthma [20,21]. Chemokines from bronchial epithelial cells have also been reported to contribute to chronic airway inflammation by recruiting inflammatory cells [22]. Eotaxins are members of the CC chemokine family, which are potent attractants of eosinophils and might contribute to airway inflammation. Eotaxins represent a group of chemokines consisting of three sets of subtypes: eotaxin-1 (CC chemokine ligand [CCL]-11) [23], eotaxin-2 (CCL24) [24], and eotaxin-3 (CCL26) [25]. Most importantly, eotaxin-3 is a more effective chemoattractant than eotaxin-1 and eotaxin-2 for eosinophils in patients with asthma [26]. RANTES (CCL5) is a chemotactic and activating factor for eosinophils, and a candidate mediator in asthma. It is able to attract several types of inflammatory cells, including eosinophils, monocytes, and T helper (Th) cells, to the site of inflammation [27]. Our current findings demonstrated that GJTWE could significantly reduce the increased production of CC chemokines, including eotaxin-3, eotaxin-1, and RANTES, in IT-stimulated BEAS-2B cells.

MMPs are enzymes that degrade the extracellular matrix and basement membrane, and regulate the infiltration of inflammatory cells; consequently, they participate in tissue remodeling [28]. MMP-9 and MMP-2, members of the gelatinase family of MMPs, are recognized to play important roles in the turnover and degradation of extracellular matrix proteins during cellular recruitment in inflammation [29]. Whereas MMP-2 is constitutively expressed in many cell types, MMP-9 is strongly induced in airway epithelial cells by inflammatory cytokines, particularly TNF-α [30,31]. Thus, the relative expression levels of MMP-9 were normalized to those of MMP-2. Our current findings indicate that GJTWE significantly decreases the MMP-9 activity in IT-stimulated BEAS-2B cells.

The upregulation of adhesion molecules on the surface of respiratory epithelial cells is an important factor in the development of asthma. The infiltration of inflammatory cells mostly results from the enhanced adhesion of leukocytes to epithelial cells via the expression of adhesion molecules [32]. Members of the immunoglobulin superfamily of endothelial adhesion molecules, ICAM-1 and VCAM-1, play an important role in inflammatory cell infiltration into inflamed airways [33]. The current results demonstrated that treatment with GJTWE significantly suppresses the increased expression of adhesion molecules, including ICAM-1 and VCAM-1, in IT-stimulated BEAS-2B cells. Taken together, the findings demonstrated that the administration of GJTWE has anti-inflammatory activity, at least via the downregulation of CC chemokine expression and MMP-9 activity, resulting in the reduced expression of adhesion molecules in IT-stimulated BEAS-2B cells.

GJT is composed of five herbal medicines, and the effects of the individual herbs on airway inflammation-related diseases, such as asthma and bronchitis, has been reported earlier. *C. cassia* protects airway epithelia from human respiratory syncytial virus [34]. *P. lactiflora* was shown to improve allergic asthma in a mouse model by inhibiting Ca^2+^ influx-dependent mast cell degranulation [35]. *G. uralensis* was reported to reduce the airway responsiveness in patients with asthma [36], and *Z. officinale* ameliorated allergic airway inflammation by suppressing the Th2-mediated immune response [37]. *Z. jujuba* also exhibits potent anti-asthmatic activity [38].

The main ingredients of GJT include phenylpropanoids (e.g., cinnamaldehyde and cinnamic acid), terpenoids (e.g., albiflorin, paeoniflorin, benzoylpaeoniflorin, and glycyrrhizin), flavonoids (e.g., liquiritin, liquiritigenin, and liquiritin apioside), chalcones (e.g., isoliquiritin and isoliquiritigenin), and phenolic compounds (e.g., 6-gingerol and 6-shogaol) [10,11]. In this study, UPLC-DAD-MS/MS analysis was performed in order to confirm the phytochemicals of GJTWE, and 21 compounds—including six flavonoids (catechin, schaftoside, isovitexin, liquiritin apioside, liquiritin, and liquiritigenin), two chalcones (isoliquiritin and isoliquiritigenin), five terpenoids (albiflorin, paeoniflorin, benzoylpaeoniflorin, glycyrrhizin, and glycyrrhetinic acid), six phenolics (protocatechuic acid, protocatechualdehyde, syringaldehyde, 1,2,3,4,6-*O*-pentagalloylglucose, 6-gingerol, and 6-shogaol), one phenylpropanoid (cinnamaldehyde), and one coumarin (coumarin)—were identified.

Several studies have previously reported the effects of various compounds contained in the herbal composition of GJT on airway inflammation-related diseases. Paeoniflorin is known to exhibit anti-asthmatic effects by inhibiting the abnormal proliferation and migration of airway smooth muscle cells, and by modulating the Th1/Th2 equilibrium [39,40]. Glycyrrhizin has been reported to reduce airway inflammation in vivo [41], and to ameliorate the progression of asthma and long-term chronic histopathological changes in the lungs of a mouse model of asthma [42,43]. In addition, jujuboside B, 6-shogaol and 6-gingerol exhibited potent anti-asthmatic effects in a murine asthma model by regulating an exaggerated inflammatory response, improving airway hyperresponsiveness, and suppressing airway inflammation [38,44,45]. The previous studies and our current data collectively suggest that the anti-inflammatory activity of GJTWE in IT-stimulated BEAS-2B cells may be due to a synergistic effect between several of the compounds of GJTWE. Further investigations are needed to clarify their mechanisms of action by identifying the phytochemicals and their contribution to the efficacy of GJTWE through quantitative analysis in detail.

In conclusion, GJTWE inhibited the production of chemokines, such as eotaxin-3, eotaxin-1 and RANTES, and reduced the MMP-9 activity and expression of adhesion molecules ICAM-1 and VCAM-1 in IT-stimulated BEAS-2B cells. Therefore, our results revealed that GJTWE might exhibit anti-inflammatory effects on airway inflammation by suppressing the expression of inflammatory response-related factors. Moreover, 21 phytochemicals were confirmed in GJTWE by UPLC-DAD-MS/MS analysis. Further studies would be required to determine the bioactive compounds that inhibit airway inflammation.

## 4. Materials and Methods

### 4.1. Materials and Reagents

The GJTWE was provided by the Herbal Medicine Research Division, Korea Institute of Oriental Medicine (Daejeon, South Korea). The detailed extraction method of GJTWE was described in a previous paper [12]. The 21 reference standards (purity > 95%) used to identify the phytochemicals in GJTWE were purchased from TargetMol (Boston, MA, USA), with the exception of liquiritin apioside, albiflorin, and protocatechuic acid (ChemFaces, Wuhan, China). LC-MS-grade water, acetonitrile, methanol, and formic acid were obtained from Thermo Fisher Scientific (Waltham, MA, USA).

### 4.2. Cell Culture

The human bronchial epithelial cell line BEAS-2B was obtained from the American Type Culture Collection (ATCC; Rockville, MD, USA). The cells were cultured in Dulbecco’s modified Eagle’s medium (DMEM; Gibco Inc., New York, NY, USA) supplemented with 10% heat-inactivated fetal bovine serum (FBS; Gibco Inc.), penicillin (100 U/mL, Gibco Inc.), and streptomycin (100 μg/mL, Gibco Inc.) at 37 °C in an atmosphere of 5% CO_2_/95% air under saturating humidity.

### 4.3. Cytotoxicity Assay

The cell viability was assessed using a cell CCK-8 assay (Dojindo, Kumamoto, Japan) according to the manufacturer’s instructions. BEAS-2B cells (6 × 10^3^ cells/well) were incubated in 96-well plates with various concentrations (31.25, 62.5, 125, 250, and 500 μg/mL) of GJTWE for 24 h. CCK-8 reagent was added to each well and incubated for 4 h. The absorbance was measured at 450 nm using a Benchmark Plus microplate reader (Bio-Rad Laboratories, Hercules, CA, USA). The percentage of cell viability was calculated using the following formula: cell viability (%) = (mean absorbance in the test sample wells/mean absorbance in the vehicle-treated control wells) × 100.

### 4.4. Cell Stimulation

BEAS-2B cells (5 × 10^5^ cells/well) were cultured in 6-well plates in a medium containing 10% FBS. After having reached confluence, the cells were washed and incubated with 1 mL serum-free medium containing 50 ng/mL IT (R&D Systems Inc., Minneapolis, MN, USA) to produce eotaxin-3, eotaxin-1, RANTES, MMPs, and adhesion molecules for 48 h.

### 4.5. Measurement of Chemokine Production

Culture supernatants were used to measure the production of eotaxin-3, eotaxin-1, and RANTES using an enzyme-linked immunosorbent assay (ELISA) protocol from R&D Systems Inc. (Minneapolis, MN, USA), according to the manufacturer’s instructions (Catalog No. DY278, DY320 and DY346). The absorbance was measured at 450 nm using a Benchmark Plus microplate reader (Bio-Rad Laboratories, Hercules, CA, USA).

### 4.6. Measurement of MMP-9 Activity

The MMP-9 activity was measured by gelatin zymography. The cell supernatant was mixed with 5× non-reducing sample buffer (Fermentas Inc., Pittsburg, PA, USA) before being loaded onto a 10% sodium dodecyl sulfate-polyacrylamide gel electrophoresis setup (SDS-PAGE; Bio-Rad Laboratories, Hercules, CA, USA) containing 1% gelatin as an MMP substrate. The samples were subjected to electrophoresis at 80 V for 2 h. Following the electrophoresis, the gels were washed twice in 2.5% Triton X-100 (Sigma-Aldrich, St. Louis, MO, USA) for 1 h to remove the SDS, and then incubated for 16 h at 37 °C in developing buffer (1 M Tris-HCl, pH 7.5, 10 mM CaCl_2_). Following incubation, the gels were stained with Coomassie Brilliant Blue G (Sigma-Aldrich, St. Louis, MO, USA) for 35 min, de-stained in 25% methanol and 8% acetic acid solution for 20 min, and finally rinsed twice with de-staining solution in order to visualize the digested bands in the gelatin matrix. The gelatinase activity was manifested as white bands on a blue background, representing areas of proteolysis of the substrate protein. The relative expression levels of the MMP-9 were normalized to those of MMP-2. Images of the gels were collected, and the average of the band intensities was measured using the commercially available ChemiDoc^TM^ XRS^+^ imaging system (Bio-Rad Laboratories, Hercules, CA, USA).

### 4.7. Measurement of the Adhesion Molecule Expression

The total ribonucleic acid (RNA) was isolated using a TRIzol reagent according to the manufacturer’s instructions (Invitrogen, Carlsbad, CA, USA). One microgram of total RNA was converted to complementary deoxyribonucleic acid (cDNA) using an iScript cDNA synthesis kit (Bio-Rad Laboratories, Hercules, CA, USA) containing oligo-dT primers, and diethyl pyrocarbonate-treated water was added to make a final volume of 20 μL; it was incubated at 42 °C for 30 min thereafter. The polymerase chain reaction (PCR)-based amplification used gene-specific primers for ICAM-1 (forward, 5′-AGG CCT TAT TCC TCC CTT CC-3′; reverse, 5′-TCA CTG CAG GAA ACT GGA GC-3′), VCAM-1 (forward, 5′-CAT TGA CTT GCA GCA CCA CA′; reverse, 5′-TCC AGC CTG TCA AAT GGG TA-3′), and GAPDH (forward, 5′-GTG ATG GCA TGG ACT GTG GT-3′; reverse, 5′-AAG GGT CAT CAT CTC TGC CC-3′). The reverse transcription (RT)-PCR reaction mixture was comprised of 1 μL cDNA and 1.56 μL γTaq PCR master mix (ELPIS biotech, Daejeon, Republic of Korea), which contained 1.5 mM MgCl_2_, 0.1 M of each forward and reverse primer, and 7.44 μL water in a final volume of 10 μL. The PCR reaction was comprised of 22 cycles of denaturation at 94 °C for 30 s, annealing at 55 °C for 1 min, and extension at 72 °C for 1 min 30 s for ICAM-1; 29 cycles of denaturation at 94 °C for 30 s, annealing at 55 °C for 1 min, and extension at 72 °C for 1 min 30 s for VCAM-1; and 25 cycles of denaturation at 94 °C for 30 s, annealing at 52 °C for 1 min, and extension at 72 °C for 1 min 30 s for GAPDH. Each reaction was performed in a Bio-Rad MyCycler™ Thermal Cycler (Bio-Rad Laboratories). The relative ratio of ICAM-1 and VCAM-1 expression was adjusted based on the expression of GAPDH as a control. This assay was performed in triplicate. The amplified products were separated on a 1.5% agarose gel and visualized using loading STAR staining (Dynebio, Seongnam, Korea). The images were captured and analyzed using the ChemiDoc^TM^ XRS^+^ imaging system (Bio-Rad Laboratories, Hercules, CA, USA).

### 4.8. UPLC-DAD-MS-MS Analysis of GJTWE

GJTWE was dissolved in methanol up to a concentration of 20 mg/mL and filtered using a syringe filter (0.2-μm pore size). The standards were prepared in methanol at a final concentration of 10 µg/mL for the UPLC-DAD-MS/MS analysis. The 21 phytochemicals in GJTWE were analyzed using a Dionex UltiMate 3000 system equipped with a Thermo Q-Exactive mass spectrometer according to the previously reported methods [46]. An Acquity BEH C_18_ column (100 × 2.1 mm, 1.7 µm, Waters Corp., Milford, MA, USA), maintained at 40 °C, was used to separate the compounds in GJT. The mobile phases consisted of 0.1% (*v*/*v*) formic acid in water (A) and acetonitrile (B). Gradient elution was performed with a flow rate of 0.25 mL/min, as follows: 0–1 min, 3% B; 1–2 min, 3–15% B; 2–13 min, 15–50% B; 13–20 min, 50–100% B; 20–23 min, 100% B; and 23.5–27.5 min, 3% B. The injection volume was 3 μL for analysis. The MS analysis was conducted with an electrospray ionization source in both positive and negative ionization modes using a Q-Exactive mass spectrometer. The MS spectra were acquired in full MS-ddMS^2^ mode. The optimized MS/MS conditions were as follows: ion spray voltage, 3.8 kV; capillary temperature, 320 °C; sheath gas pressure, 40 arbitrary units (au); auxiliary gas pressure, 10 au; S-lens RF level, 60; resolution, 70000 (full MS) and 17500 (ddMS^2^); scan range, 100–1500 m/z; and normalized collision energy, 25 eV. All of the data were acquired and processed using Xcalibur v.3.0 and TraceFinder v.3.2 software (Thermo Fisher Scientific, Bremen, Germany).

### 4.9. Statistical Analyses

All of the data are presented as the mean ± standard error of the mean (SEM). The statistical significance was determined using analysis of variance (ANOVA) followed by Dunnett’s multiple comparisons test. Statistical significance was set at *p* < 0.05 or < 0.01.

## Figures and Tables

**Table 1:** Phytochemicals identified from GJTWE by UPLC-DAD-MS/MS.

No.	Rt ^1^ (min)	Calculated (*m*/*z*)	Measured (*m*/*z*)	Adduct	Error (ppm)	Formula	MS/MS (*m*/*z*)	Identifications
1	4.32	153.0193	153.0187	[M−H]^−^	−3.9699	C_7_H_6_O_4_	153.0176, 109.0276	Protocatechuic acid [16]
2	5.09	139.0390	139.0389	[M + H]^+^	−0.5419	C_7_H_6_O_3_	139.0388, 121.0285, 111.0443, 93.0340	Protocatechualdehyde [16]
3	5.16	289.0718	289.0710	[M−H]^−^	3.5430	C_15_H_14_O_6_	289.0709, 245.0809, 203.0701, 179.0491, 165.0176, 137.0228	Catechin [17]
4	5.93	563.1406	563.1392	[M−H]^−^	−2.5017	C_26_H_28_O_14_	563.1381, 473.1062, 443.0975, 383.0745, 353.0655	Schaftoside [17]
5	6.04	481.1704	481.1698	[M + H]^+^	−1.3305	C_23_H_28_O_11_	179.0700, 151.0752, 133.0648	Albiflorin [17]
6	6.28	525.1614	525.1634	[M + HCO_2_]^−^	3.8676	C_23_H_28_O_11_	327.1074, 165.0541, 121.0277	Paeoniflorin [17]
7	6.67	433.1129	433.1126	[M + H]^+^	−0.7161	C_21_H_20_O_10_	433.1061, 415.1018, 397.0916, 379.0807, 351.0856, 313.0702	Isovitexin [19]
8	6.73	549.1614	549.1637	[M−H]^−^	4.2543	C_26_H_30_O_13_	549.1605, 417.1173, 255.0652, 135.0069, 119.0487	Liquiritin apioside [17]
9	6.85	183.0652	183.0652	[M + H]^+^	−0.1077	C_9_H_10_O_4_	183.0652, 123.0442	Syringaldehyde [16]
10	6.87	417.1191	417.1182	[M−H]^−^	−2.2678	C_21_H_22_O_9_	417.1183, 255.0653, 135.0070, 119.0483	Liquiritin [17]
11	7.01	939.1109	939.1087	[M−H]^−^	−2.3388	C_41_H_32_O_26_	769.0862, 617.0756, 447.0557, 295.0450, 169.0126	1,2,3,4,6-*O*-Pentagalloylglucose [17]
12	8.65	417.1191	417.1183	[M−H]^−^	4.0974	C_21_H_22_O_9_	417.1176, 255.0652, 135.0070, 119.0481	Isoliquiritin [17]
13	8.80	147.0441	147.0439	[M + H]^+^	−0.9875	C_9_H_6_O_2_	147.0439, 103.0547, 91.0548, 77.0394, 65.0394	Coumarin [16]
14	9.28	255.0663	255.0656	[M−H]^−^	−2.7953	C_15_H_12_O_4_	255.0651, 135.0069, 119.0483	Liquiritigenin [17]
15	10.84	629.1876	629.1862	[M + HCO_2_]^−^	−2.1643	C_30_H_32_O_12_	431.1359, 165.0540, 121.0276	Benzoylpaeoniflorin [17]
16	11.14	133.0648	133.0648	[M + H]^+^	−0.0089	C_9_H_8_O	133.0647, 115.0544, 105.0702, 103.0546, 91.0548, 79.0550, 77.0394, 55.0188	Cinnamaldehyde [16]
17	12.02	255.0663	255.0671	[M−H]^−^	3.1272	C_15_H_12_O_4_	255.0652, 153.0180, 135.0069, 119.0483	Isoliquiritigenin [17]
18	13.18	821.3965	821.3999	[M−H]^−^	4.1300	C_42_H_62_O_16_	821.3943, 351.0556, 193.0343	Glycyrrhizin [17]
19	14.52	317.1723	317.1720	[M + Na] ^+^	−0.9535	C_17_H_26_O_4_	317.1690, 299.1064	6-Gingerol [18]
20	17.68	277.1798	277.1809	[M + H]^+^	4.0361	C_17_H_24_O_3_	277.2159, 259.2050, 137.0596	6-Shogaol [18]
21	19.02	471.3469	471.3463	[M + H]^+^	−1.2879	C_30_H_46_O_4_	471.3464, 407.3315, 317.2107, 189.1635	Glycyrrhetinic acid [17]

^1^ Rt: retention time (min).

## Data Availability

The data presented in this study are available within the article.
